# Comparing the Effectiveness of the Blended Delivery Mode With the Face-to-Face Delivery Mode of Smoking Cessation Treatment: Noninferiority Randomized Controlled Trial

**DOI:** 10.2196/47040

**Published:** 2024-02-20

**Authors:** Lutz Siemer, Marcel E Pieterse, Somaya Ben Allouch, Marloes G Postel, Marjolein G J Brusse-Keizer

**Affiliations:** 1 School of Social Work Saxion University of Applied Sciences Enschede Netherlands; 2 Department of Psychology, Health and Technology Centre for eHealth & Well-being Research - Behavioural, Management and Social Sciences University of Twente Enschede Netherlands; 3 Research Group Technology, Health & Care Saxion University of Applied Sciences Enschede Netherlands; 4 Digital Life Research Group Amsterdam University of Applied Science Amsterdam Netherlands; 5 Digital Interactions Lab (DIL) Informatics Institute University of Amsterdam Amsterdam Netherlands; 6 Medical School Twente Medisch Spectrum Twente Enschede Netherlands; 7 Health Technology & Services Research Technical Medical (TechMed) Centre University of Twente Enschede Netherlands

**Keywords:** tobacco, blended treatment, smoking cessation, randomized controlled trial, effectiveness, noninferiority, evaluation, mobile phone

## Abstract

**Background:**

Tobacco consumption is a leading cause of death and disease, killing >8 million people each year. Smoking cessation significantly reduces the risk of developing smoking-related diseases. Although combined treatment for addiction is promising, evidence of its effectiveness is still emerging. Currently, there is no published research comparing the effectiveness of blended smoking cessation treatments (BSCTs) with face-to-face (F2F) treatments, where web-based components replace 50% of the F2F components in blended treatment.

**Objective:**

The primary objective of this 2-arm noninferiority randomized controlled trial was to determine whether a BSCT is noninferior to an F2F treatment with identical ingredients in achieving abstinence rates.

**Methods:**

This study included 344 individuals who smoke (at least 1 cigarette per day) attending an outpatient smoking cessation clinic in the Netherlands. The participants received either a blended 50% F2F and 50% web-based BSCT or only F2F treatment with similar content and intensity. The primary outcome measure was cotinine-validated abstinence rates from all smoking products at 3 and 15 months after treatment initiation. Additional measures included carbon monoxide–validated point prevalence abstinence; self-reported point prevalence abstinence; and self-reported continuous abstinence rates at 3, 6, 9, and 15 months after treatment initiation.

**Results:**

None of the 13 outcomes showed statistically confirmed noninferiority of the BSCT, whereas 4 outcomes showed significantly (*P*<.001) inferior abstinence rates of the BSCT: cotinine-validated point prevalence abstinence rate at 3 months (difference 12.7, 95% CI 6.2-19.4), self-reported point prevalence abstinence rate at 6 months (difference 19.3, 95% CI 11.5-27.0) and at 15 months (difference 11.7, 95% CI 5.8-17.9), and self-reported continuous abstinence rate at 6 months (difference 13.8, 95% CI 6.8-20.8). The remaining 9 outcomes, including the cotinine-validated point prevalence abstinence rate at 15 months, were inconclusive.

**Conclusions:**

In this high-intensity outpatient smoking cessation trial, the blended mode was predominantly less effective than the traditional F2F mode. The results contradict the widely assumed potential benefits of blended treatment and suggest that further research is needed to identify the critical factors in the design of blended interventions.

**Trial Registration:**

Netherlands Trial Register 27150; https://onderzoekmetmensen.nl/nl/trial/27150

**International Registered Report Identifier (IRRID):**

RR2-doi.org/10.1186/s12889-016-3851-x

## Introduction

### Tobacco’s Global Impact

According to the World Health Organization [1], the tobacco epidemic is one of the biggest public health threats the world has ever faced: tobacco kills up to half of its consumers, which means >8 million people every year. Of these, >7 million deaths are because of direct tobacco consumption, whereas approximately 1.2 million are because of the exposure of nonsmokers to passive smoking [1]. The economic costs of tobacco consumption are considerable and include significant health costs of treating the disease caused by tobacco consumption and the loss of human capital through the morbidity and mortality attributable to tobacco consumption [1]. Smoking addiction is more prevalent in specific, often susceptible subpopulations, such as individuals in lower education or socioeconomic groups [2]. Approximately 80% of the world’s 1.1 billion smokers live in low- and middle-income countries, where the burden of tobacco-related diseases and deaths is the highest [1].

### Smoking Cessation Progress

People who stop smoking greatly reduce their risk of disease and early death [3] and will have major immediate and long-term health benefits [4,5]. Among smokers who are aware of the dangers of tobacco and the benefits of quitting, most want to quit [1]. Compared with quitting without professional support, smoking cessation treatment can more than double the success rates of quitting attempts [1]; this ultimately results—for treatments comparable with those in this study—in estimated point prevalence abstinence rates of 28.4% (95% CI 21.3-35.5) for treatments with a total amount of contact time of 91 to 300 minutes and of 24.7% (95% CI 21.0-28.4) for treatments with >8 person-to-person treatment sessions (both intention-to-treat [ITT]; 6 months after the quit date [6]). Previous research in the hospital smoking cessation clinic where this study was conducted showed a 19% abstinence rate for comparable treatment in the target group of patients with chronic obstructive pulmonary disease at the 12-month follow-up [7].

### Blended Treatment Evolution

In the past decades, a variety of effective interventions for smoking cessation have become available [8,9], including, more recently, eHealth services such as web-based interventions [10,11] or mobile phone–based interventions [12,13]. At present, traditional face-to-face (F2F) interventions, on the one hand, and both web-based and mobile phone–based interventions, on the other hand, are increasingly being developed into blended treatments. This development is consistent with the idea that blended treatment combines the *best of both worlds* [14,15], as the strengths of one type of treatment should compensate for the weaknesses of the other [14-21]. For example, personal attention from a professional in F2F treatment could compensate for the lack of personal contact in web-based treatment. In turn, one of the main features of web-based treatment is the possibility of being available anytime and anywhere, which could bridge the interval between sessions in F2F treatment.

A systematic review of randomized controlled trials (RCTs) by Erbe et al [17] on blended F2F and web-based interventions suggests that compared with stand-alone F2F therapy, blended therapy may save clinician time and lead to lower dropout rates and higher abstinence rates in patients with substance abuse. The authors concluded that for common mental health disorders, blended interventions are feasible and can be more effective than no-treatment controls, but more RCTs on the effectiveness of blended treatments compared with nonblended treatments are necessary.

### Objectives

Although promising, evidence available for blended deaddiction treatment is still emerging, addressing abuse of substances such as alcohol [22], cocaine, marijuana [23], or opioids [24]. For smoking cessation, we only found studies on the promising adjunctive use of smartphone apps with F2F contact [25-27]. To the best of our knowledge, there is no published research on the effectiveness of blended smoking cessation treatments (BSCTs) compared with F2F treatments, where in the blended treatment, the web-based components are not an adjunct but a substitute for specific F2F treatment components. Therefore, in this study, we present the results of an RCT comparing a blended 50% F2F and 50% web-based smoking cessation treatment with a traditional F2F treatment that was similar in content and intensity. The primary objective was to determine if a BSCT resulted in noninferior abstinence rates compared with an F2F treatment with identical ingredients. The rationale for choosing a noninferiority design was that we expected secondary benefits for the BSCT, such as lower costs, lower dropouts, and higher patient satisfaction, even if the BSCT only led to comparable abstinence rates.

## Methods

### Design

This study reports the results of an unblinded 2-arm, parallel group, noninferiority RCT with 1:1 allocation using stratified randomization (nicotine dependency, internet skills, and quitting strategy).

### Setting

The study was conducted at the outpatient smoking cessation clinic (Dutch: *Stoppen met Roken Poli*) of the Medical Spectrum Twente Hospital in Enschede, the Netherlands. Enschede is a municipality and city in the east of the Netherlands with a population of 150,000 inhabitants. The estimated daily smoking prevalence in Enschede was 17.2% in 2017, which is approximately the same as the average (17.4%) in the Netherlands, which is one of the countries with the least number of smokers in Europe [28].

### Trial Registration

The trial was registered in the Netherlands Trial Register on March 24, 2015 (Acronym: LiveSmokefree-Study; Title: Blended Smoking Cessation Treatment; new ID: NL5975; old ID: NTR5113), and a detailed protocol has been published previously [29].

### Participants

We recruited participants between March 2015 and March 2019. The participants were self-referred to the treatment or were referred to the clinic by their general practitioner or hospital physician and were called by members of the research department to check for eligibility. Eligible participants were current daily smokers (eg, at least 1 cigarette, cigar, pipe, or e-cigarette per day [30]), those who were aged ≥18 years, those who had access to the internet (eg, email and websites), and those who were able to read and write Dutch. Eligible patients completed a questionnaire at the beginning of the study before being randomized.

### Ethical Considerations

Consistent with the Dutch Medical Research Ethics Committee guidelines, this study was approved by the accredited Medical Research Ethics Committee Twente (P14-37/NL50944.044.14) and subsequently by the Board of Directors of Medisch Spectrum Twente Hospital. Before initiation, the trial was registered and a detailed protocol has been published previously [29].

A patient information letter outlining the burden of participation was distributed to all patients, and eligible patients attended an intake interview and signed a consent form.

We processed participants’ personal data in accordance with the Dutch Personal Data Protection Act. The data were collected in 2 ways as follows:

1. The data of the personal contacts were recorded on data collection forms and collected centrally at Medisch Spectrum Twente Hospital. The data manager of the study recorded all collected data in an Access 2007 (Microsoft Corp) database.
2. Most data were collected by Tactus Addiction Treatment, a regional addiction care organization with expertise in web-based treatment, using web-based questionnaires offered to both treatment groups.

Individual patients and caregivers had a log-in with a username and password secured by the Secure Sockets Layer. All data transferred between the patient’s PC and the application were encrypted and sent using the https protocol. All data were encrypted and stored on servers in secure data centers in the Netherlands. To further ensure data security, daily backups of the server were performed.

The participants did not receive any compensation for their participation in the study.

### Interventions

The study interventions to be compared were a blended F2F treatment and web-based BSCT and an F2F treatment. Except for the differences in the mode of delivery (ie, F2F mode and web mode), both treatments had the same features as follows:

1. High-intensity treatment that comprised 10 sessions (20-minute contact time for each session, except for the first session, which lasts 50 minutes) and supportive pharmacotherapy, if needed, within a 6-month period with an expected quit date after about 3 months
2. Delivered by health care professionals in an outpatient cessation clinic
3. Concordant with the Dutch guidelines for tobacco addiction [31], fulfilling the requirements of the Dutch care module for smoking cessation [32]
4. Executed by counselors registered in the Dutch quality register of qualified smoking cessation counselors
5. Supporting 3 quitting strategies that patients could choose at the start of the treatment: (1) stop at once, (2) change gradually by increasing the number of daily activities that are performed smoke free, or (3) decrease smoking at regular intervals (eg, scheduled smoking reduction by 100%->75% and 75%->50%). The chosen quitting strategy did not generally influence the course of the treatment. The order, pace, duration, and intensity were the same for all strategies.

Both BSCT and F2F treatment covered 52 behavior change techniques (using behavior change technique taxonomy v1 of 93 hierarchically clustered techniques by Michie et al [33]) as shown in Table 1.

**Table 1:** Behavior change techniques included in the BSCT^a^ and F2F^b^ treatment.

Grouping	Behavior change techniques
1. Goals and planning	1.1. Goal setting (behavior) 1.2. Problem-solving 1.3. Goal setting (outcome) 1.4. Action planning 1.5. Review behavior goal or goals 1.6. Discrepancy between current behavior and goal 1.8. Behavioral contract 1.9. Commitment
2. Feedback and monitoring	2.3 Self-monitoring of behavior 2.4 Self-monitoring of outcome or outcomes of behavior 2.6 Biofeedback 2.7 Feedback on outcome or outcomes of behavior
3. Social support	3.1 Social support (unspecified)
4. Shaping knowledge	4.2 Information about antecedents 4.3 Reattribution
5. Natural consequences	5.1 Information about health consequence 5.2 Salience of consequences 5.3 Information about social and environmental consequences 5.4 Monitoring of emotional consequences 5.5 Anticipated regret 5.6 Information about emotional consequences
6. Comparison of behavior	6.2 Social comparison 6.3 Information about others’ approval
7. Associations	7.4 Remove access to the reward
8. Repetition and substitution	8.1 Behavioral practice and rehearsal 8.2 Behavior substitution 8.3 Habit formation 8.4 Habit reversal 8.6 Generalization of a target behavior 8.7 Graded tasks
9. Comparison of outcomes	9.1 Credible source 9.2 Pros and cons 9.3 Comparative imagining of future outcomes
10. Reward and threat	10.7 Self-incentive 10.9 Self-reward
11. Regulation	11.1 Pharmacological support 11.2 Reduce negative emotions
12. Antecedents	12.1 Restructuring the physical environment 12.2 Restructuring the social environment 12.3 Avoidance or reducing exposure to cues for the behavior 12.4 Distraction
13. Identity	13.1 Identification of self as role model 13.2 Framing and reframing 13.5 Identity associated with changed behavior
14. Scheduled consequences	14.4 Reward approximation 14.5 Rewarding completion 14.6 Situation-specific reward 14.7 Reward incompatible behavior 14.8 Reward alternative behavior
15. Self-belief	15.1 Verbal persuasion about capability 15.3 Focus on past success
16. Covert learning	16.3 Vicarious consequences

^a^BSCT: blended smoking cessation treatment.

^b^F2F: face-to-face.

F2F treatment consisted of 10 F2F sessions delivered at an outpatient smoking cessation clinic. BSCT consisted of 5 F2F sessions at the outpatient clinic and 5 web-based sessions delivered via the web-based treatment platform Roken De Baas (which translates loosely as “in control of smoking”). During the RCT, the software had to be revised once, as the European General Data Protection Regulation became enforceable from May 25, 2018, which changed the appearance and handling but not the content of the interventions.

Both F2F treatment and BSCT consisted of counselor-dependent and counselor-independent components. The counselor-dependent web-based components of BSCT were interactive and relied on asynchronous communication (eg, email and SMS text messaging) between the counselor and patient. The counselor-independent components such as psychoeducational content or the smoking diary were used by the patients on their own and at their own time. In F2F treatment, these components were provided in a paper manual that clients took home. In BSCT, these components were accessible over the web. As such, both treatments were equivalent in terms of content and intensity. However, an additional benefit of BSCT was that the content of previous counselor-dependent components remained accessible as email and SMS text messaging correspondence saved on the web.

The characteristic feature of BSCT is an equal balance between F2F and web-based sessions, and the focus of the treatment was not supposed to be on the F2F mode or the web mode; in addition, there was a constantly alternating and interacting use of the F2F mode and web mode. Table 2 presents the order, timing, main features, duration, and modes of delivery of the treatment sessions for F2F treatment and BSCT. Although an even distribution was planned for BSCT with regard to the number of sessions, there was an uneven distribution for the duration of treatment because the first session (50 minutes of F2F mode) was longer than the remaining sessions (20 minutes of F2F mode or 20 minutes of web mode); therefore, BSCT patients spent 130 minutes in the F2F mode and 100 minutes in the web mode.

**Table 2:** Characteristics of the treatment sessions in F2F^a^ treatment and BSCT^b^ according to treatment protocol.

Session	Week	Main features	Duration (N=230 minutes)	Mode of delivery of treatment sessions	
				BSCT (130 minutes of F2F mode^c^ and 100 minutes of web mode^d^)	F2F treatment (230 minutes of F2F mode)
1	1	Goal setting, prompt smoking diary, and measure CO^e^ level	50	F2F	F2F
2	3	Measures for self-control	20	Web	F2F
3	5	Dealing with withdrawal	20	F2F	F2F
4	7	Breaking habits	20	Web	F2F
5	9	Dealing with triggers	20	F2F	F2F
6	11	Food for thought	20	Web	F2F
7	14	Think differently and measure CO level and cotinine^f^ level	20	F2F	F2F
8	18	Do differently	20	Web	F2F
9	22	Action plan and measure CO level	20	F2F	F2F
10	26	Closure	20	Web	F2F

^a^F2F: face-to-face.

^b^BSCT: blended smoking cessation treatment.

^c^F2F mode: F2F sessions of BSCT.

^d^Web mode: web-based sessions of BSCT.

^e^CO: carbon monoxide.

^f^Cotinine measurement was only performed in patients who reported quitting smoking either in the 3-month follow-up questionnaire or during treatment to the counselor.

More information about both treatments can be found in the study protocol of the RCT [29] and in the description of the user experiences of BSCT [21]. The treatment fidelity of the counselors was not recorded. The adherence to the treatments was described elsewhere [34,35]; but, in brief, levels of adherence were comparable for BSCT and F2F treatment sessions. To provide an impression of the look and feel of the web interventions of BSCT, Multimedia Appendix 1 displays screenshots of the web-based sessions of BSCT.

### Outcomes

For the primary objective (ie, effectiveness) of the analysis, the primary outcome for the ITT analysis of the treatments’ effectiveness in smoking cessation was the proportions of biochemically (ie, cotinine) validated point prevalence abstinence from all combustible tobacco products (eg, cigarettes, bags, cigars, and pipes) at 3 and 15 months after the start of the treatment. Additional outcomes were the proportions of carbon monoxide (CO)–validated point prevalence abstinence; self-reported point prevalence abstinence; and self-reported continuous abstinence at 3 (ie, shortly after the expected quit date), 6 (ie, end of treatment), and 9 and 15 (follow-up measurements) months. Applying the noninferiority margin justified in our protocol paper [29], BSCT was considered as noninferior if it resulted in abstinence rates that were <5% points lower than those of F2F treatment [29].

### Measurements

#### Effectiveness

Cotinine-validated and CO-validated abstinence measurements were used to measure biochemically validated point prevalence abstinence rates [36-38].

Cotinine measurement was performed at approximately the 3-month and 15-month follow-up (ie, shortly after the expected quit day, week 14; refer to Table 2) and at the 15-month follow-up only in patients who reported quitting either during the treatment to the counselor or in the 3-month or 15-month follow-up questionnaire. A 0.5 mL to 1 mL salivary sample was collected using a Salivette (Sarstedt AG and Co). Under supervision, patients chew on a cotton swab for 1 minute to stimulate the saliva flow rate. All saliva specimens were frozen until assayed and transported to the laboratory for the determination of cotinine levels using a gas chromatography technique. Abstinence was defined as having a salivary cotinine level <20 ng/mL [39].

The CO level was measured in all patients (independent of reporting quitting) at 3 months, at the last F2F treatment session at the hospital (for the BSCT group, the last F2F treatment session was at 5 months after the start of the treatment [week 22], and for the F2F treatment group, at 6 months after start of the treatment [week 26]; refer to Table 2), and in patients who reported quitting at 15 months together with the cotinine level. A breath CO level of 5 ppm was taken as the cutoff value between smokers and nonsmokers (≥5 ppm in smokers and <5 ppm in nonsmokers [40]). Breath CO levels were monitored using a piCO Smokerlyzer (Bedfont Instruments), a portable CO monitor.

Furthermore, self-reported point prevalence abstinence and self-reported continuous abstinence rates were measured at 3, 6, 9, and 15 months after treatment initiation. The measurement tool was a standardized questionnaire for Dutch tobacco research [30], which patients in both BSCT and F2F treatment completed over the web. Self-reported point prevalence abstinence rate was assessed by asking patients whether they had smoked ≥1 cigarette (eg, bags, cigars, and pipe) in the last 7 days, and the self-reported continuous abstinence rate was assessed by asking whether they had smoked since the current stop.

For each measurement during and after treatment, the participants were prompted twice via email and, in the absence of measurements, were additionally notified twice via telephone. If no measurement was available after 2 emails and 2 telephone calls, the participants were classified as lost to follow-up for the respective measurement and notified again for the next measurement.

#### Sample Size

For the RCT, we calculated the abstinence rates for 344 participants, assuming a long-term abstinence rate of 10% for those receiving F2F treatment [6,7,41] and—based on its expected benefits—15% for those receiving BSCT. If BSCT would lead to an abstinence rate not <5%, it would be considered as noninferior compared with F2F treatment. Therefore, 172 patients per group with a power of 80% and a Cronbach α of .025 were needed for this RCT (calculated using Power Analysis & Sample Size [NCSS Statistical Software]).

### Randomization

We randomly allocated patients to either BSCT or F2F treatment using computerized randomization (Qminim Online Minimization). Randomization was performed at the individual level (allocation ratio 1:1). The minimization was stratified according to (1) the level of internet skills [42], (2) the level of nicotine dependence [30], and (3) the quitting strategy favored by the patient (eg, stop at once, gradual change, and scheduled reduced smoking; for details refer to the description in the *Interventions* section). The data used for minimization were collected using the baseline questionnaire, which was completed over the web by the patient after providing consent.

### Blinding

Owing to the nature of the treatment conditions, it was self-evidently impossible to blind the staff and patients involved in the study.

### Statistical Methods

For both the BSCT group and the F2F treatment group, the patients’ demographic, smoking-related, and health-related characteristics at baseline were reported as means with SD for normally distributed continuous variables and as medians with IQR for nonnormally distributed continuous variables. Categorical variables were reported as numbers with corresponding percentages. To identify between-group differences, an independent 1-sided (1-tailed) *t* test or Mann-Whitney *U* test was performed as appropriate for continuous variables, and Pearson chi-square or Fisher exact test was performed for categorical variables.

As this was an ITT analysis, participants with missing data on smoking status were considered as smokers. The absolute and proportional abstinence rates in the treatment group were reported.

The noninferiority was analyzed by calculating the difference and the 95% CI of the observed difference in the abstinence rates and by comparing that to the previously defined noninferiority margin of 5% points [29]. In addition, the noninferiority analysis is illustrated in a forest chart.

To be able to compare the results of this study with those of other studies conducted using a more traditional RCT design, additional repeated measures analyses were conducted using generalized estimating equation to test for group, time, and group×time differences in abstinence rates.

All analyses were performed using the SPSS software (version 26.0; IBM Corp), except for the calculation of the CIs of the difference between abstinence rates, for which we used the web tool by VassarStats [43] for “The Confidence Interval For The Difference Between Two Independent Proportions.”

## Results

### Participant Flow

**Figure 1:**
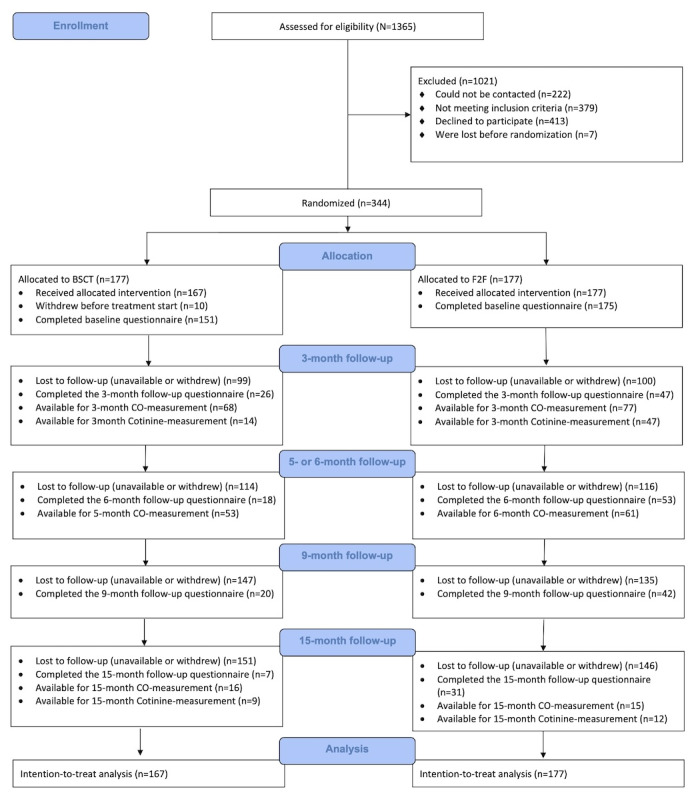
Flow of participants through the study. Withdrawals are noncumulative. BSCT: blended smoking cessation treatment; CO: carbon monoxide; F2F: face-to-face.

Figure 1 shows the flow of participants throughout the study. A total of 344 patients were eligible for the study, provided written consent, and were randomized (smoking cessation treatment: BSCT, n=177; F2F treatment, n=177). Of 177 patients each in both groups, 167 (94.3%) patients of the BSCT group and all 177 (100%) of the F2F treatment group started treatment (ie, they received at least 1 session). Before the start of treatment, 151 (85.3%) of the 177 patients in the BSCT group and 175 (98.8%) of the 177 patients in the F2F treatment group completed the baseline questionnaire. Three months after starting treatment (ie, shortly after the expected quit date), of the 177 patients in the BSCT group, 14 (7.9%) who self-reported quitting were available for cotinine measurement, 68 (38.4%) were available for CO measurement, and 26 (14.6%) completed the follow-up questionnaire. Of the 177 patients in the F2F treatment group, 47 (26.5%) who reported quitting were available for cotinine measurement, 77 (43.5%) were available for CO measurement, and 47 (26.5%) completed the 3-month follow-up questionnaire. Of 177 patients in the BSCT group, 53 (29.9%) were available for the 5-month CO measurement and 18 (10.1%) completed the 6-month follow-up questionnaire. Of 177 patients in the F2F treatment group, 61 (34.4%) were available for the 6-month CO measurement and 53 (29.9%) completed the 6-month follow-up questionnaire. The 9-month follow-up questionnaire was completed by 20 (11.2%) patients of the BSCT group and 42 (23.7%) patients of the F2F treatment group. After 15 months of starting treatment, 9 (5.1%) of the 177 patients in the BSCT group who self-reported quitting were available for cotinine level measurement. A total of 16 (9%) patients in the BSCT group were available for CO level measurement and 7 (4%) completed the follow-up questionnaire. Of 177 patients in the F2F treatment group, 12 (6.8%) patients who reported quitting were available for cotinine level measurement, 15 (8.5%) were available for CO level measurement, and 31 (17.5%) completed the 15-month follow-up questionnaire.

### Baseline Characteristics

Table 3 shows that the baseline characteristics, including demographic, smoking-related, and health-related characteristics, were comparable between the participants in both groups.

**Table 3:** Patients’ characteristics of the BSCT^a^ group and the F2F^b^ treatment group.

Characteristics				BSCT		F2F treatment
**Demographics**						
	**Sex (BSCT: n=152; F2F: n=175), n (%)**					
		Female	77 (50.7)		87 (49.7)	
		Male	75 (49.3)		88 (50.3)	
	**Nationality (BSCT: n=152; F2F: n=175), n (%)**					
		Dutch	147 (96.7)		173 (98.9)	
		Other	5 (3.3)		2 (1.1)	
	**Cultural background (BSCT: n=152; F2F: n=175), n (%)**					
		Dutch	137 (90.1)		161 (92)	
		Other	15 (9.9)		14 (8)	
	Age (y; BSCT: n=152; F2F: n=175), mean (SD)			47.5 (12.4)		46.4 (13.2)
	**Marital status (BSCT: n=151; F2F: n=175), n (%)**					
		With partner	99 (65.6)		107 (61.1)	
		Alone	52 (34.4)		68 (38.9)	
	**Housing situation (BSCT: n=152; F2F: n=172), n (%)**					
		With children	63 (41.5)		73 (42.4)	
		Without children	89 (58.6)		99 (57.6)	
	**Education (BSCT: n=152; F2F: n=171), n (%)**					
		VET^c^ or higher	96 (63.1)		109 (63.7)	
		Lower than VET	56 (36.8)		62 (36.3)	
	**Main income (BSCT: n=152; F2F: n=175), n (%)**					
		Wage or own company	79 (52)		89 (50.9)	
		Income support	73 (48)		86 (49.1)	
	**Main day activity (BSCT: n=152; F2F: n=175), n (%)**					
		Paid work	77 (50.7)		85 (48.6)	
		Other	75 (49.3)		90 (51.4)	
	Internet skills^d^ (BSCT: n=151; F2F: n=175), mean (SD)			38.4 (6.5)		39.4 (5.8)
**Smoking-related characteristics**						
	**Quitting strategy (BSCT: n=167; F2F: n=176), n (%)**					
		Stop at once	68 (40.7)		72 (40.9)	
		Change gradually	44 (26.4)		44 (25)	
		Scheduled reduction	55 (32.9)		60 (34.1)	
	**Reason to start treatment (BSCT: n=152; F2F: n=175), n (%)**					
		Intrinsic	104 (68.4)		117 (66.9)	
		Extrinsic	48 (31.6)		58 (33.1)	
	Nicotine dependency (Fagerström^e^; BSCT: n=151; F2F: n=175), mean (SD)			5.3 (2.1)		5.2 (2.1)
	Negative attitude toward quitting^f^ (BSCT: n=151; F2F: n=175), mean (SD)			−5.5 (3.1)		−5.1 (2.9)
	Positive attitude toward quitting^g^ (BSCT: n=151; F2F: n=175), median (IQR)			10 (8-11)		10 (9-11)
	Self-efficacy^h^ (BSCT: n=151; F2F: n=175), mean (SD)			−0.2 (5.2)		−0.4 (4.9)
	Readiness to quit^i^ (BSCT: n=151; F2F: n=175), median (IQR)			2 (1-3)		2 (1-3)
	Earlier quit attempts (BSCT: n=151; F2F: n=175), n (%)			128 (84.8)		154 (88)
	Social support^j^ (BSCT: n=151; F2F: n=175), median (IQR)			4 (3-4)		4 (3-5)
	Social modeling^k^ (BSCT: n=151; F2F: n=175), median (IQR)			3 (1-6)		3 (1-5)
	Use of alcohol^l^ (BSCT: n=151; F2F: n=175), median (IQR)			2 (1-3)		2 (1-3)
	Use of (recreational) drugs (BSCT: n=151; F2F: n=175), n (%)			11 (7.3)		15 (8.6)
**Health-related characteristics**						
	Use of medication in general (BSCT: n=151; F2F: n=175), n (%)			102 (67.6)		130 (74.3)
	Use of medication for addiction treatment (BSCT: n=151; F2F: n=175), n (%)			0 (0)		0 (0)
	Use of medication for psychiatric treatment (BSCT: n=151; F2F: n=159), n (%)			30 (19.9)		26 (16.4)
	Use of medication for physical treatment (BSCT: n=151; F2F: n=159), n (%)			78 (51.7)		93 (58.5)
	Use of other medication (BSCT: n=151; F2F: n=159), n (%)			25 (16.6)		31 (19.5)
	Health-related complaints^m^ (MAP HSS^n^; BSCT: n=151; F2F: n=175), mean (SD)			12.6 (6.2)		12.6 (6.6)
	Smoking-related complaints^o^ (BSCT: n=151; F2F: n=175), mean (SD)			21.0 (13.6)		20.8 (9.2)
	Health- and smoking-related complaints^p^ (BSCT: n=151; F2F: n=175), mean (SD)			33.6 (13.6)		33.4 (14.2)
	Depression^q^ (BSCT: n=151; F2F: n=175), median (IQR)			4 (0-10)		4 (2-12)
	Anxiety^q^ (BSCT: n=151; F2F: n=175), median (IQR)			4 (2-8)		6 (2-10)
	Stress^q^ (BSCT: n=151; F2F: n=175), median (IQR)			8 (4-16)		10 (4-16)
	DASS^r^ (BSCT: n=151; F2F: n=175), median (IQR)			18 (8-32)		20 (8-36)
	EQ-5D-3L^s^ (BSCT: n=151; F2F: n=175), median (IQR)			0.8 (0.7-1.0)		−0.8 (0.7-0.9)
	EQ VAS^t^ (BSCT: n=151; F2F: n=175), mean (SD)			65.5 (18.7)		64.6 (18.7)

^a^BSCT: blended smoking cessation treatment.

^b^F2F: face-to-face.

^c^VET: vocational education and training.

^d^Internet skills: range 10-60; higher numbers indicate better skills.

^e^Nicotine dependency (Fagerström): range 0-10; higher numbers indicate higher nicotine dependency.

^f^Negative attitude toward quitting: range −12 to 0; lower numbers indicate a more negative attitude toward quitting smoking.

^g^Positive attitude toward quitting: range 0-12; higher numbers indicate a more positive attitude toward quitting smoking.

^h^Self-efficacy: range −12 to 12; higher numbers indicate higher self-efficacy related to smoking cessation.

^i^Readiness to quit: range 0-4; higher numbers indicate higher readiness to quit.

^j^Social support: range 0-5; higher numbers indicate more social support in smoking cessation.

^k^Social modeling: range 0-8; higher numbers indicate more smokers in the social environment.

^l^Use of alcohol: range 0-4; 0=Never, 1=1 time per month, 2=2-4 times per month, 3=2-3 times per week, and 4=≥4 times per week.

^m^Health-related complaints: range 0-40; higher numbers indicate poorer health status.

^n^MAP HSS: Maudsley Addiction Profile Health Symptoms Scale.

^o^Smoking-related complaints: range 0-64; higher numbers indicate more smoking-related complaints.

^p^Health- and smoking-related complaints: range 0-104; higher numbers indicate poorer health status and more smoking-related complaints.

^q^Depression, anxiety and stress: range 0-42; higher numbers indicate a higher level of depression, anxiety and stress.

^r^DASS: sum score of depression, anxiety and stress (range 0-126; higher numbers indicate a more negative emotional status).

^s^EQ-5D-3L: societal-based quantification of the patients’ health status (range 0-1; higher numbers indicate better health status).

^t^EQ VAS: visual analog scale for quality of life (range 0-100, higher numbers indicate better state of health).

### Effectiveness

Table 4 shows the results of effectiveness measurements at 3, 5 or 6, 9, and 15 months after the start of treatment. The cotinine-validated point prevalence abstinence shortly after the expected stop day (ie, 3 months after the treatment initiation) showed a significantly lower and inferior abstinence rate in the BSCT group (4.8%) than in the F2F treatment group (17.5%; difference of 12.7, 95% CI 6.2-19.4; *P*<.001). The differences found in the 15-month cotinine level measurement (difference of 1.5, 95% CI −3.5 to 6.4) and in all CO level measurements at 3 months (difference of 2.5, 95% CI −6.9 to 11.8), 5 or 6 months (difference 3.7, 95% CI −4.0 to 11.4), and 15 months (difference 0.7, 95% CI −4.9 to 6.7) were not substantial and inconclusive in terms of inferiority.

**Table 4:** Treatment effects on the participants in both BSCT^a^ (n=167) and F2F^b^ treatment (n=177) groups at 3, 5 or 6, 9, and 15 months after the start of treatment.

Outcome		3 months	5 or 6 months	9 months	15 months
**Cotinine-validated point prevalence abstinence**					
	F2F treatment, n (%)	31 (17.5)	—^c^	—	10 (5.7)
	BSCT, n (%)	8 (4.8)	—	—	7 (4.2)
	Percentage points, difference (95% CI)	12.7 (6.2 to 19.4)	—	—	1.5 (−3.5 to 6.4)
**CO^d^-validated point prevalence abstinence**					
	F2F treatment, n (%)	50 (28.2)	31 (17.5)	—	13 (7.3)
	BSCT, n (%)	43 (25.7)	23 (13.8)	—	11 (6.6)
	Percentage points, difference (95% CI)	2.5 (−6.9 to 11.8)	3.7 (−4.0 to 11.4)	—	0.7 (−4.9 to 6.7)
**Self-reported point prevalence abstinence^e^**					
	F2F treatment, n (%)	35 (19.8)	48 (27.1)	39 (22)	26 (14.7)
	BSCT, n (%)	22 (13.2)	13 (7.8)	19 (11.4)	5 (3)
	Percentage points, difference (95% CI)	6.6 (−1.3 to 14.4)	19.3 (11.5 to 27.0)	10.7 (2.8 to 18.4)	11.7 (5.8 to 17.9)
**Self-reported continuous abstinence^f^**					
	F2F treatment, n (%)	30 (16.9)	35 (19.8)	23 (13)	20 (11.3)
	BSCT, n (%)	19 (11.4)	10 (6)	13 (7.8)	3 (1.8)
	Percentage points, difference (95% CI)	5.6 (−1.9 to 13.0)	13.8 (6.8 to 20.8)	5.2 (−1.4 to 11.8)	9.5 (4.4 to 15.1)

^a^BSCT: blended smoking cessation treatment.

^b^F2F: face-to-face.

^c^Data not available.

^d^CO: carbon monoxide.

^e^Answer “no” to the questionnaire question “Have you smoked one or more cigarettes (bags, cigars, pipe) in the last 7 days?”

^f^Answer “no” to the questionnaire question “Have you smoked since the stop?”

Furthermore, we observed significantly lower and inferior abstinence rates in the BSCT group for self-reported point prevalence abstinence at 5 or 6 months (BSCT 7.8% vs F2F treatment 27.1%; difference 19.3, 95% CI 11.5-27.0; *P*<.001), for self-reported point prevalence abstinence at 15 months (BSCT 3% vs F2F treatment 14.7%; difference 11.7, 95% CI 5.8-17.9; *P*<.001), and for self-reported continuous abstinence at 5 or 6 months (BSCT 6% vs F2F treatment 19.8%; difference 13.8, 95% CI 6.8-20.8; *P*<.001). Significantly lower—but in terms of inferiority, inconclusive—abstinence rates in the BSCT group were found for self-reported point prevalence abstinence at 9 months (BSCT 11.4% vs F2F treatment 22%; difference 10.7, 95% CI 2.8-18.4; *P*=.009) and for self-reported continuous abstinence at 15 months (BSCT 1.8% vs F2F treatment 11.3%; difference 9.5, 95% CI 4.4-15.1; *P*<.001).

**Figure 2:**
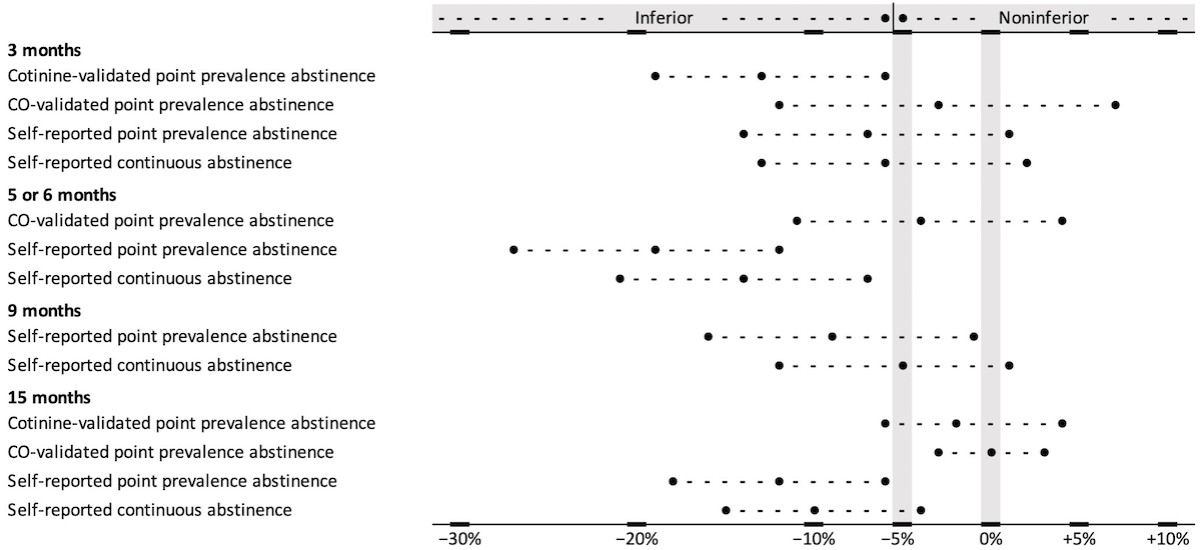
Forest plot of the risk differences between the blended smoking cessation treatment (BSCT; 95% CI) and face-to-face (F2F) treatment. CO: carbon monoxide.

Figure 2 presents the 95% CIs of the differences between BSCT and F2F treatment groups for all abstinence outcome measures by applying the 5% points noninferiority margin. The forest plot illustrates the inferiority of BSCT with cotinine-validated point prevalence abstinence at 3 months, self-reported point prevalence abstinence at 6 and 15 months, and self-reported continuous abstinence at 6 months. For the remaining outcomes, the forest plot shows inconclusive results.

The generalized estimating equation analysis showed significant differences (*P*<.05) between both the groups with time and the group×time interaction for cotinine-validated point prevalence abstinence, self-reported point prevalence abstinence, and self-reported continuous abstinence rates. For the CO-validated point prevalence abstinence, a significant difference was found with time, but neither was there a difference between the groups nor a time×group interaction.

## Discussion

### Principal Findings

This paper presents the results of an RCT comparing the effectiveness of a blended 50% F2F treatment and 50% web-based BSCT to F2F-only treatment with similar ingredients and intensity. Contrary to our expectations, the abstinence rates of the BSCT group were lower than those of the F2F group. For the primary outcome (ie, cotinine-validated point prevalence abstinence rate), applying the 5%-point noninferiority margin indicated inferiority of BSCT at 3 months, whereas the outcome at 15 months was inconclusive. Both results should be considered with caution as the statistical power to detect differences was limited owing to nonresponse. Furthermore, BSCT was found to be inferior in 3 of the secondary outcomes (ie, self-reported point prevalence abstinence rate, self-reported continuous abstinence rate at 6 months, and self-reported point prevalence abstinence rate at 15 months), whereas the remaining outcomes were inconclusive. Although most outcomes from the repeated measures analyses showed significantly lower abstinence rates for the blended treatment, all remaining outcomes were nonsignificant, further corroborating the inferiority of BSCT against F2F treatment.

Given that our results suggest that it is more likely that BSCT is inferior to F2F treatment, our study is not consistent with the higher abstinence rates reported in the literature [17] for blended treatments compared with F2F treatment. Explanations for this likely inferiority of BSCT require further study. As the patients’ demographic, smoking-related, and health-related characteristics were comparable in both treatment groups, these factors did not seem to play a role in this context. This also applies to adherence; as we found in previous analyses [35], adherence was comparable for both the groups. However, we know from qualitative analyses conducted as part of this RCT [21] that participants found the web-based components of BSCT to be rather unmotivating and not enjoyable, which may have resulted in BSCT patients making less use of the web-based components both during and after the treatment, and thus, may be a factor in the lower abstinence rates. The experience of patients in the BSCT suggests that the highly protocolized, equally balanced mix for blended treatment chosen in this study, with a fixed sequence of alternating F2F and web-based sessions, was too restrictive for blended treatment in practice [35], thus limiting tailoring to individual patient needs. Which intervention components should be offered when and in what form to achieve optimal treatment outcomes requires further investigation.

Furthermore, even if this cannot be supported by systematic observations and analyses, we believe that provider-related factors at the microlevel (eg, the treatment fidelity of counselors and therapist drift) and at the mesolevel (eg, the organization’s preexisting knowledge, routines, and leadership) should be considered more closely. A relevant factor could be that, in the development of BSCT, half of the counseling sessions of the F2F treatment established in the outpatient smoking cessation clinic were replaced by web-based tools from a web platform unfamiliar to the clinic and counselors. Therefore, counselors had half of their F2F intervention replaced and had to integrate the new web-based components into a new blended workflow. The preexisting routine and familiarity with the F2F treatment among counselors might have disadvantaged the quality of execution of the blended treatment. The normalization process theory [44] could provide valuable perspectives in this context. It posits that the unclear definition of BSCT’s meaningfulness (coherence) for counselors may have diminished their motivation and engagement (cognitive participation). Limited collective agency in BSCT’s implementation, owing to rigid protocols and insufficient reflective monitoring, may have further impeded its establishment in clinical practice. Understanding these barriers through targeted investigations could enhance the integration and efficacy of the BSCT.

Although not the focus of this analysis, we noticed that both treatments mostly showed lower abstinence rates than those reported in the literature for comparable treatments (ie, point prevalence abstinence rates of 28.4% for treatments with a total contact time of 91 to 300 minutes 6 months after the quit date [6]). At 9 months (ie, 6 months after the quit date), for F2F treatment, we found a self-reported point prevalence abstinence rate of 22% and a self-reported continuous abstinence rate of 13%. For BSCT, this rate was even lower with 11.4% for self-reported point prevalence abstinence and 7.8% for self-reported continuous abstinence. These relatively low abstinence rates could be because of the population characteristics (ie, patients in an outpatient smoking cessation clinic in a hospital context). Further analysis should investigate whether the sample differs from the general population in terms of known effectiveness predictors [45], such as, in this context, age, socioeconomic status, alcohol and drug use, health status, nicotine dependence, motivation to quit, or family status. However, a previous study by Christenhusz [7] in the same clinic with a comparable treatment for the specific target group of patients with chronic obstructive pulmonary disease showed much higher cotinine-validated abstinence rates (19%) compared with F2F treatment (5.7%) and BSCT (4.2%) at the 12-month follow-up. A more plausible explanation for this is the high dropout rate and missing data in this study. As we applied the common penalized imputation procedure (assuming missing=smoking [46,47]) to deal with missing data in our analyses, imputed quit rates will decrease proportionally to dropout rates.

A final point to consider is that toward the end of the RCT, the software of the web platform had to be updated because of legal changes, which temporarily caused accessibility problems. However, as only a few patients were affected by this and only toward the end of the RCT, these had no relevant influence on the results of this study.

Although blended treatment appears promising and reflects today’s digitalization of the lifestyle of patients and health care professionals [14,15], not every realization of blended treatment is automatically an improvement. This also underscores the need to answer the question Greenhalgh et al [48] raised earlier: “What explains the success of a blended treatment in one context and the failure of a comparable blended treatment in another context?” The likely inferiority of BSCT in this study indicates that the current realization of BSCT will have to be reconsidered, which may involve aspects such as the optimal balance and mix of F2F and web-based components or the use of synchronous versus asynchronous counseling within web-based components. Such a redesign process can be supported by an analysis using the normalization process theory [44] and guided by an eHealth development model such as the Center for eHealth Research Road map [49]; the nonadoption, abandonment, scale-up, spread, and sustainability framework [48]; or more practically by the “Fit for Blended Care” instrument [15], which is intended to support therapists and patients in deciding whether and how blended care can be established.

For the generalization of the results, it should be noted that this analysis referred to a hospital context and a blended treatment with a strict 50:50 ratio of web-based and F2F interventions. For example, hospital patients could be expected to have a higher disease burden and, possibly, owing to age, a lower eHealth literacy than the general population. The question arises whether the results would have been different in a healthier, younger population. In addition, as mentioned above, a fixed 50:50 ratio of web-based and F2F interventions was defined for BSCT, which did not consider the individual needs of patients or counselors. A blended treatment that is better tailored to the needs, characteristics, and skills of both the patients and the counselors could have led to better results [15]. We know from an earlier study by Siemer et al [21] that patients would have preferred to use a smartphone app instead of a web platform, for example, or that they would have liked to be free to choose the ratio and sequence of F2F and web-based interventions.

### Limitations

A major limitation of this study was the high dropout rate at several follow-up time points, resulting in many missing values for both self-reported and biochemical measures, which had a major impact on the ITT analysis. According to the ITT procedure, all missing values for the outcomes were coded as smoking. As a result, both the biochemically validated outcomes and the self-reported outcomes of this study are likely to be overly conservative, which largely explains the relatively low abstinence rates found in both study groups compared with the existing literature.

Furthermore, because of the high dropout rate, we conducted an analysis of the factors associated with dropout. We identified 2 main predictors of dropout: having a smoking partner at baseline and lower mental health scores as indicated by the Depression Anxiety Stress Scale [50]. Both predictors are known to be associated with poorer treatment outcomes [51]. This finding suggests that neither of the interventions used in this study sufficiently reduced the barriers to successful intervention completion. Such nonrandom patterns of dropout pose a threat to the external validity of our findings as they suggest that our sample may not be fully representative of the wider population. However, it is notable that these attrition factors alone are unlikely to fully explain the relatively low quit rates observed in this trial compared with other similarly intensive interventions [7] as similar reasons for dropout are likely to occur in any smoking cessation trial sample. Nevertheless, the underrepresentation of participants with these risk factors at later follow-ups may have led to an overestimation of the effectiveness of our interventions, although it remains difficult to assess whether this occurred to a greater extent in this study than in other smoking cessation trials.

However, the most critical aspect is whether these predictors of attrition varied between the 2 treatment conditions [52]. Such differential attrition could compromise the internal validity of our findings, particularly in the noninferiority test comparing blended treatment with F2F treatment delivery. Owing to low cell counts of the two above-mentioned dropout predictors, a detailed analysis to examine the interaction effects of attrition predictors by treatment condition was not feasible. In addition, there were no consistent differences in the attrition rates between the study groups at any follow-up time points.

Another limitation to consider is the risk of bias in patient-reported outcome measures (PROMs), such as social desirability or recall bias, particularly given that the study relies in part on self-reported smoking cessation for an extended period of 15 months. The participants’ ability to accurately recall their smoking behavior could be impaired, especially in the context of continuous abstinence, which could bias the study results. In general, the lower quit rates in our study compared with the existing literature argue against a significant self-report bias, as PROMs tend to overestimate quit rates compared with biochemical validation. Furthermore, biased PROMs will only have affected the internal validity of this study if they occur differently in the 2 study groups. We have no indications of this, but we lack data to verify this statistically. Because the determination of noninferiority is ultimately based on applying the 5% margin, it can also be considered a weakness that this 5% margin is based only on our considerations, as stated in the protocol paper of this study [29]. However, a slightly higher or lower margin would have led to slight changes in the results but not fundamental changes in the conclusions.

In addition to the cotinine measurements, this study collected CO measurements at the last 3 follow-up points. However, the second of these CO measurements showed a 4-week difference between the groups: 5 months after baseline for the blended treatment group and 6 months for the F2F group. Assuming that relapse rates would be expected to increase with time, this difference should have favored the effectiveness of the blended treatment. However, our results at this time point show the opposite, further supporting our claim of inferiority of the blended treatment compared with the F2F treatment.

Another limitation of this study is that our data, which were designed to compare the 2 approaches of blended and F2F treatment, did not allow analyses at the level of treatment components within the 2 delivery modes. Nevertheless, studies comparing different blended protocols are warranted to enable the design of improved BSCT in the future.

A final limitation of this study is that, as is often the case in clinical studies [53], we have not recorded the treatment fidelity and therefore deviations from the treatment protocol favoring one of both modes of delivery may have biased our findings. Although we cannot rely on systematic observations, we have some reason to believe that the implementation and adoption of the innovative BSCT may have had a negative impact on the effectiveness of the BSCT compared with the usual F2F treatment. However, based on our data on adherence from previous papers [34,35] and the findings on satisfaction (not reported in this study), we found no indication of a fidelity issue.

### Conclusions

In this analysis of an RCT comparing a BSCT with a comparable F2F treatment, we found predominant results indicating inferiority of the blended mode compared with the traditional F2F mode, exceeding a 5% margin in abstinence rate. This could not be explained by lower adherence. Further research is required on the critical factors involved in the design of blended interventions.

## Appendix

Multimedia Appendix 1 Screenshots of web sessions of the blended smoking cessation treatment.

Multimedia Appendix 2 CONSORT eHEALTH checklist.

## Abbreviations

BSCT: blended smoking cessation treatment
CO: carbon monoxide
F2F: face-to-face
ITT: intention-to-treat
PROM: patient-reported outcome measure
RCT: randomized controlled trial

## Data Availability

The data underlying this paper are available in Data Archiving and Networked Services [57].
